# Discovery of stable and prognostic CT-based radiomic features independent of contrast administration and dimensionality in oesophageal cancer

**DOI:** 10.1371/journal.pone.0225550

**Published:** 2019-11-22

**Authors:** Concetta Piazzese, Kieran Foley, Philip Whybra, Chris Hurt, Tom Crosby, Emiliano Spezi

**Affiliations:** 1 School of Engineering, Cardiff University, Cardiff, United Kingdom; 2 Velindre Cancer Centre, Cardiff, United Kingdom; 3 Centre for Trials Research, Cardiff, United Kingdom; Chang Gung Memorial Hospital at Linkou, TAIWAN

## Abstract

The aim of this work was to investigate radiomic analysis of contrast and non-contrast enhanced planning CT images of oesophageal cancer (OC) patients in terms of stability, dimensionality and contrast agent dependency. The prognostic significance of CT-based radiomic features was also evaluated. Different 2D and 3D radiomic features were extracted from contrast and non-contrast enhanced CT images of 213 patients from the multi-centre SCOPE1 randomised controlled trial (RCT) in OC. Feature stability was evaluated by randomly dividing patients into three groups and identifying textures with similar distributions among groups with a Kruskal-Wallis analysis. A paired two-sided Wilcoxon signed rank test was used to assess for significant differences in the remaining corresponding 2D and 3D stable features. A prognostic model was constructed using clinical characteristics and remaining filtered features. The discriminative ability of significant variables was tested using Kaplan-Meier analysis. A total of 238 2D and 3D radiomic features were computed from oesophageal CT images. More than 75 features were stable if extracted from homogeneous cohort (contrast or non-contrast enhanced CT images) and inhomogeneous cohort (contrast and non-contrast enhanced CT images). Among the remaining corresponding stable features computed from both cohorts, only 4 features did not show a statistically significant difference if obtained in 2D or in 3D (p-value < 0.05). A Cox regression model constructed using 5 clinical variables (age, sex, tumour, node and metastasis (TNM) stage, WHO performance status and contrast administration) and 4 radiomic variables (inverse variance_GLCM_, large distance emphasis_GLDZM_, zone distance non uniformity norm_GLDZM_, zone distance variance_GLDZM_), identified one radiomic feature (zone distance variance_GLDZM_) that was significantly associated with overall survival (p-value = 0.032, HR = 1.25, 95% CI = 1.02–1.52). A significant difference in overall survival between groups was found when considering a threshold of zone distance variance_GLDZM_ equals to 1.70 (X^2^ = 7.692, df = 1, p-value = 0.006). Zone distance variance_GLDZM_ was identified as the only stable CT radiomic feature statistically correlated with overall survival, independent of dimensionality and contrast administration. This feature was able to identify high-risk patients and if validated, could be the subject of a future clinical trial aiming to improve clinical decision making and personalise OC treatment.

## 1. Introduction

Oesophageal cancer (OC) is the eighth most common malignancy worldwide with a 5-year overall survival rate between 15% and 25% [1]. If patients are deemed to have potentially curable disease, a combination of chemotherapy, radiotherapy and surgery is used, depending on their tumour, node and metastasis (TNM) stage, physiological fitness and personal choice[2, 3]. Despite these treatment options, many patients still have a poor prognosis, suggesting that current treatment efficacy is suboptimal and clinical decision making can be improved to better select which treatment to use for each patient.

Radiological staging largely informs the likely patient prognosis [4], so techniques that identify prognostic imaging biomarkers from staging investigations may therefore improve subsequent clinical treatment decisions. A non-invasive approach could assist the risk-stratification of patients with OC, by identifying groups of patients that may respond to treatment, or those that are likely to suffer side-effects but with little benefit to prognosis.

Radiomics is a new field that has gained increasing attention in recent years [5]. A large number of quantitative features can be extracted from medical images, including parameters not appreciated by simple visual analysis, which could improve the prediction of patient outcomes [6]. Radiomics have been investigated in OC [7, 8]and could be used to inform future decision support systems [9].

Several studies have focussed on evaluating sensitivity, repeatability and reproducibility of radiomics features from different imaging modalities and anatomical regions [10, 11, 12, 13]. The prognostic and predictive value of CT-based radiomics have previously been evaluated in OC. In the work of Ganeshan et al. [14], the prognostic value of texture analysis was assessed in non-contrast enhanced CT images, whereas contrast enhanced CT images were used to predict response to chemoradiotherapy (CRT) in Hou et al. [15] and Nakajo et al. [16]. However, common limitations of currently published works are the often small sample sizes used to perform radiomic analyses and the lack of stability testing of image features across heterogenous datasets.

A common example of variation in a radiological dataset is the presence or absence of intravenous (I.V.) contrast in CT examinations. Ideally, staging CT examinations should be performed with I.V. contrast to improve detection of the tumour, lymph nodes and metastases, and to aid treatment planning. However, some patients are unable to have I.V. contrast due to poor renal function or allergy [17]. Administration of I.V. contrast is often desired in clinical trials although not a pre-requisite for inclusion. Radiomic features that are stable across contrast versus non-contrast enhanced CT images and 2-dimensional (2D) versus 3-dimensional (3D) images would overcome this limitation and increase their application across multiple datasets.

Accordingly, the first aim of this study was to evaluate the stability, dimensionality and contrast agent dependency of radiomic features extracted from contrasted and non-contrast CT examinations of patients with OC, acquired during the multi-centre SCOPE1 randomised controlled trial (RCT). The second aim of the work was to evaluate the prognostic significance of discovered stable radiomic features as potential future imaging biomarkers in personalised OC staging.

## 2. Materials and methods

### 2.1. Population and CT imaging

This study retrospectively included a cohort of 213 patients (with radiotherapy planning CT) recruited into the SCOPE1 trial [18], a National Cancer Research Institute and Cancer Research UK funded Phase II/III two arm clinical trial investigating definitive CRT with and without cetuximab in OC. The SCOPE1 trial (EUDRACT 2006-002241-37; ISRCTN 4771849) was ethically approved by the Research Ethics Committee for Wales on 17/04/2007. The trial was performed in accordance with the study protocol and monitored by the trial management group. All patients recruited to SCOPE1 provided written informed consent prior to randomisation and treatment initiation. Patients included in the trial were also informed of the strict confidentiality of their data and the possible review and use by authorised individuals other than their treating physician [19]. Detailed baseline characteristics of subjects enrolled in this study are presented in Table 1.

**Table 1:** Baseline characteristics of the clinical cohort.

		Frequency (%)
Median age		73 (range 42–90)
Gender (M:F)		119 (55.9) : 94 (44.1)
Histology	Adenocarcinoma	53 (24.9)
	Squamous cell carcinoma	156 (73.2)
	Undifferentiated carcinoma	4 (1.9)
Tumour location	Upper third	23 (10.8)
	Middle third	98 (46.0)
	Lower third	92 (43.2)
Contrast agent	Yes	138 (64.8)
	No	75 (35.2)
WHO performance status	0	110 (51.6)
	1	103 (48.4)
Stage group	I	8 (3.8)
	II	41 (19.2)
	III	129 (60.6)
	IV	35 (16.4)
Treatment	Control arm (CRT)	108 (50.7)
	Research arm. (CRT + cetuximab)	105 (49.3)
Overall survival	Alive	128 (60.1)
	Dead	85 (39.9)

Patients underwent CT scan examination in different hospitals in UK. Oesophageal contrast-enhanced and non-contrast enhanced CT data were obtained using different scanners (Siemens, GE medical systems, Philips) and the following acquisition parameters: 120 kV; 20–641 mAs; reconstruction diameter, 361–700; matrix, 512 × 512; pixel spacing, 0.71–1.37 mm; slice thickness, 2.5–5 mm; time between injection and CT, 35-40s.

On each 2D axial CT image, the gross tumour volume (GTV) was manually outlined following the SCOPE1 protocol by an expert oncologist. The CERR software package [20] was used to process and import the CT images and radiotherapy volumes. The cohort was stratified into groups of patients with contrasted (n = 138) and non-contrasted CT (n = 75).

### 2.2. Radiomic features extraction

Using an in-house developed data analytics software [21], radiomic features were automatically calculated in compliance with the Image Biomarker Standardisation Initiative (IBSI) [22], an international collaboration aiming to standardise radiomic information extracted from medical images in order to perform high-throughput quantitative image analysis. Before extracting the features, CT images were isotropically resampled to 2 mm resolution in all three directions using linear interpolation. The list of extracted second-order and high-order texture features included: grey level co-occurrence matrix (GLCM), grey level run length matrix (GLRL), grey level size zone matrix (GLSZM), grey level distance zone matrix (GLDZM), neighbourhood grey tone difference matrix (NGTDM). Second-order and high-order features were obtained considering one segment layer at a time (2D) or considering the whole tumour volume (3D). Furthermore, GLCM and GLRLM values were calculated by considering two types of aggregation methods. Texture features were obtained “without merging” when considering each 2D directional matrix and averaging over 2D directions and slices, or when considering each 3D directional matrix and averaging over the 3D directions. Features were calculated “with merging” when the radiomic value was calculated from a single matrix after merging all 2D directional matrices or when considering a single matrix after merging all 3D directional matrices. The list of radiomic features extracted for each oesophageal tumour is summarized in Table 2.

**Table 2:** Radiomic features extracted from contrast and non-contrast enhanced CT images. GLCM, grey level co-occurrence matrix; GLRLM, grey level run length matrix; GLSZM, grey level size zone matrix; GLDZM, grey level distance zone matrix; NGTDM, neighbourhood grey tone difference matrix.

Texture type	Dimension	Summarising features	Feature computed
GLCM	2D/3D	with/without merging	100
GLRLM	2D/3D	with/without merging	64
GLSZM	2D/3D	-	32
GLDZM	2D/3D	-	32
NGTDM	2D/3D	-	10

### 2.3. Radiomic features selection and stability comparison

**Fig 1:**
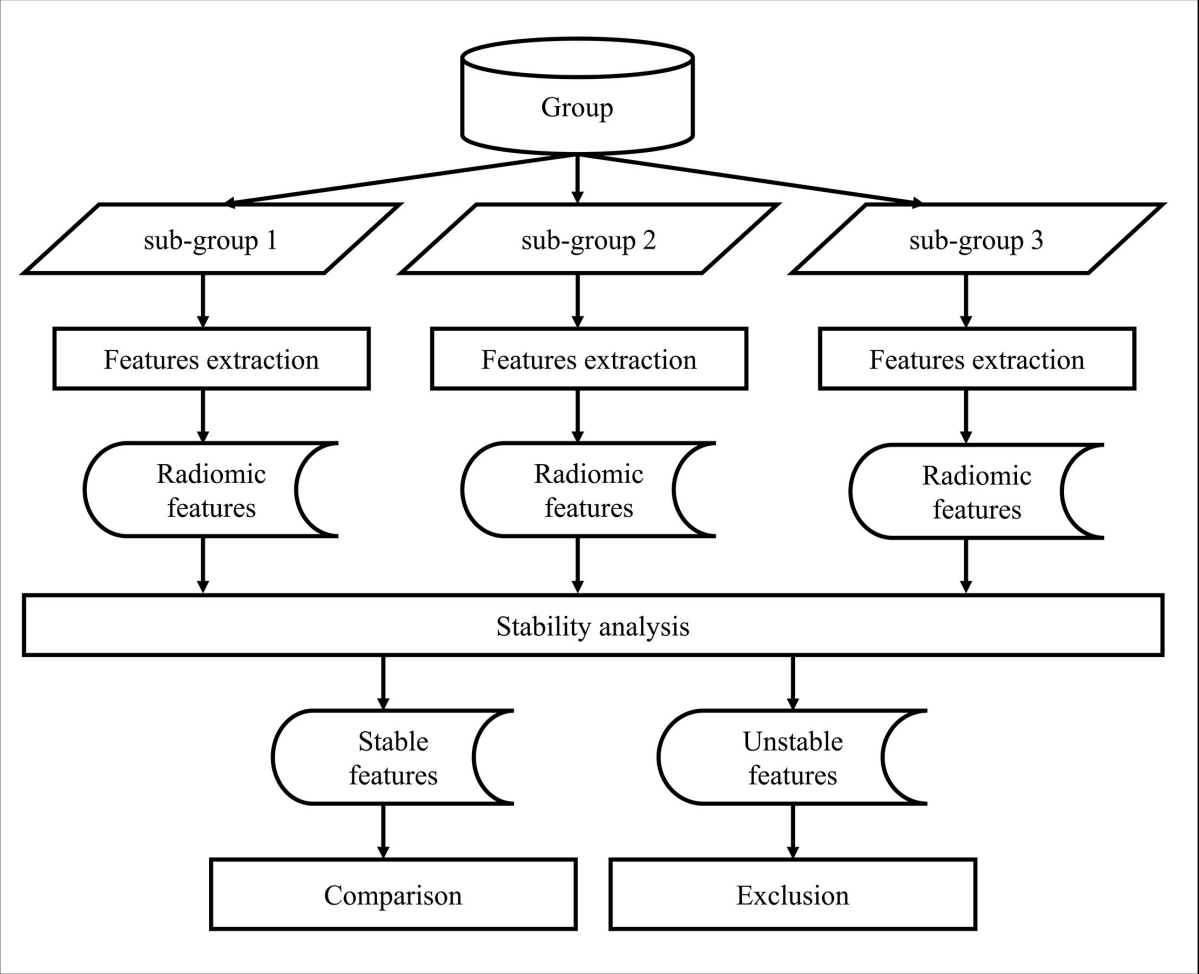
Workflow used for selecting stable radiomic features. The workflow was repeated three times using three different groups (a “mixed group” with contrast and non-contrast enhanced CT images, a “contrast group” with contrast enhanced CT images and a “non-contrast group” with non-contrast enhanced CT images) as input data. The input group was divided in three sub-groups (n = 71 for the mixed group, n = 46 for the contrast group and n = 25 for the non-contrast group) and processed to extract the features. Stable features, identified as the ones with similar distributions among the sub- groups, were further analysed. Feature with different distributions among sub-group (identified as unstable) were not further investigated.

To investigate the possible impact of contrast agents on texture-based features extracted from CT images of OC, three different groups of patients were analysed: “mixed group” with contrast and non-contrast enhanced CT scans (n = 213), “contrast group” cohort with CT images acquired with I.V. contrast (n = 138) and “non-contrast group” with CT images acquired without I.V. contrast (n = 75). Each group was used as input in the workflow depicted in Fig 1. Three sub-groups were created by randomly dividing the clinical cohort of patients using the function “randi” of Matlab (Matlab 2017b; Mathworks, Natick, MA, USA) that generates uniformly distributed random integers in a specified interval. From each sub-group, features were extracted in 2D, in 3D, with and without merging. Features with similar distributions among sets were identified as stable using the Kruskal-Wallis test. Features with different distributions were identified as unstable and excluded from further analysis. The remaining corresponding 2D and 3D stable features within each group were compared to assess for significant differences with the paired two-sided Wilcoxon signed rank test. Kruskal-Wallis and paired two-sided Wilcoxon signed rank test were performed using Matlab. For both tests, a p-value of <0.05 was considered statistically significant.

For each group, stable features that showed no difference if obtained considering one segment layer at a time in 2D or the whole tumour layers in 3D were grouped and evaluated with intraclass correlation coefficient (ICC). Features resembling each other (ICC > 0.90) were removed.

Remaining radiomic features were further investigated to evaluate their potential prognostic value. A Cox regression model was constructed using 5 clinical variables (age, sex, TNM stage, WHO performance status and contrast administration) and stable radiomic features. The Cox regression was computed with a backward conditional method, which has previously been recommended [23] and used in published studies [8]. Survival comparison was performed with the Kaplan-Meier life-table method. In particular, the independent cohort of patients enrolled in this study was divided into three groups equally populated by using prognostic variables obtained from the Cox regression model. A log-rank test evaluated significant differences in overall survival (OS). Differences with a p value of < 0.05 were considered statistically significant. Survival analysis was performed using SPSS Statistics version 23.0 (IBM, Chicago, IL, USA).

## 3. Results

**Fig 2:**
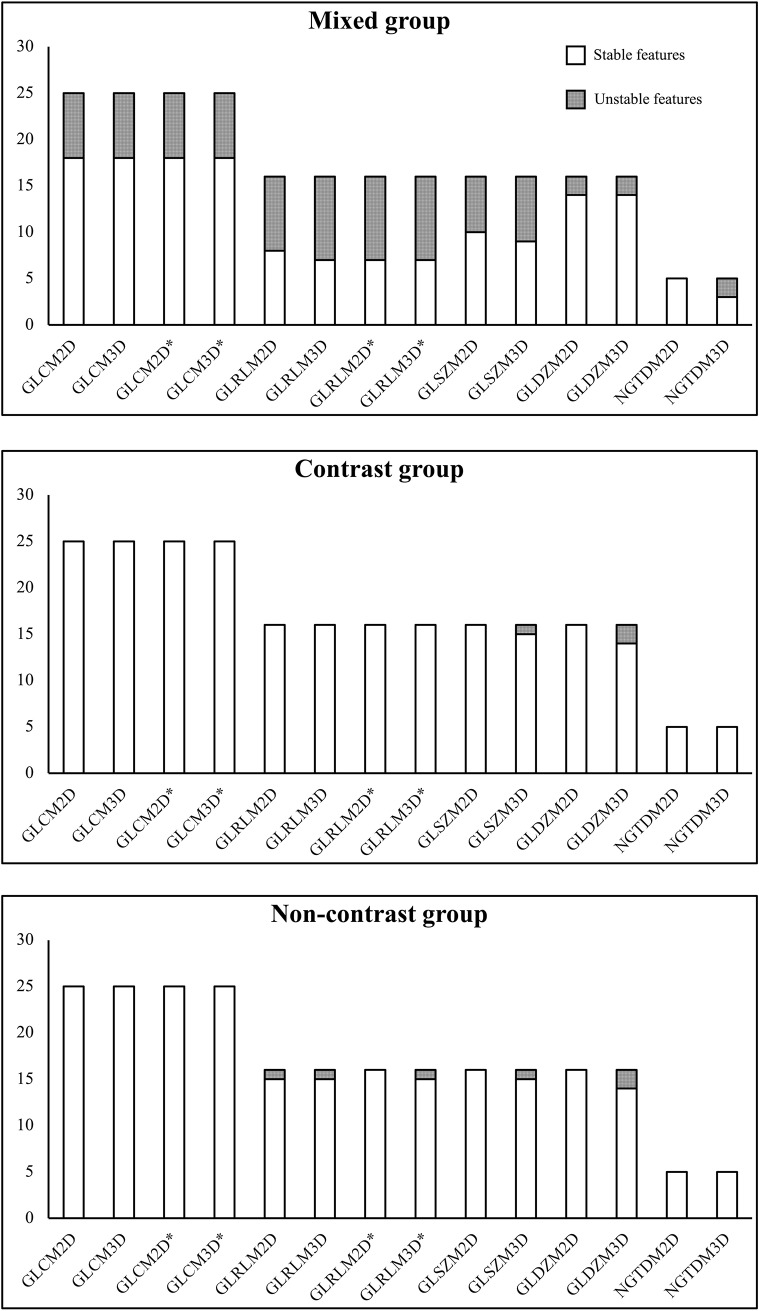
Stable and unstable radiomic features extracted from the three groups. GLCM, grey level co-occurrence matrix; GLRLM, grey level run length matrix; GLSZM, grey level size zone matrix; GLDZM, grey level distance zone matrix; NGTDM, neighbourhood grey tone difference matrix; *, feature computed with merging.

A total of 238 2D and 3D radiomic features were extracted from the CT images of the oesophagus. Fig 2 shows both stable and unstable radiomic features extracted from the three groups. The majority of the features were stable in the three sub- groups. Conversely, unstable features were identified with the Kruskal-Wallis test in the mixed group (n = 82), contrast group (n = 3) and non-contrast group (n = 6), respectively, and excluded from further analysis.

**Fig 3:**
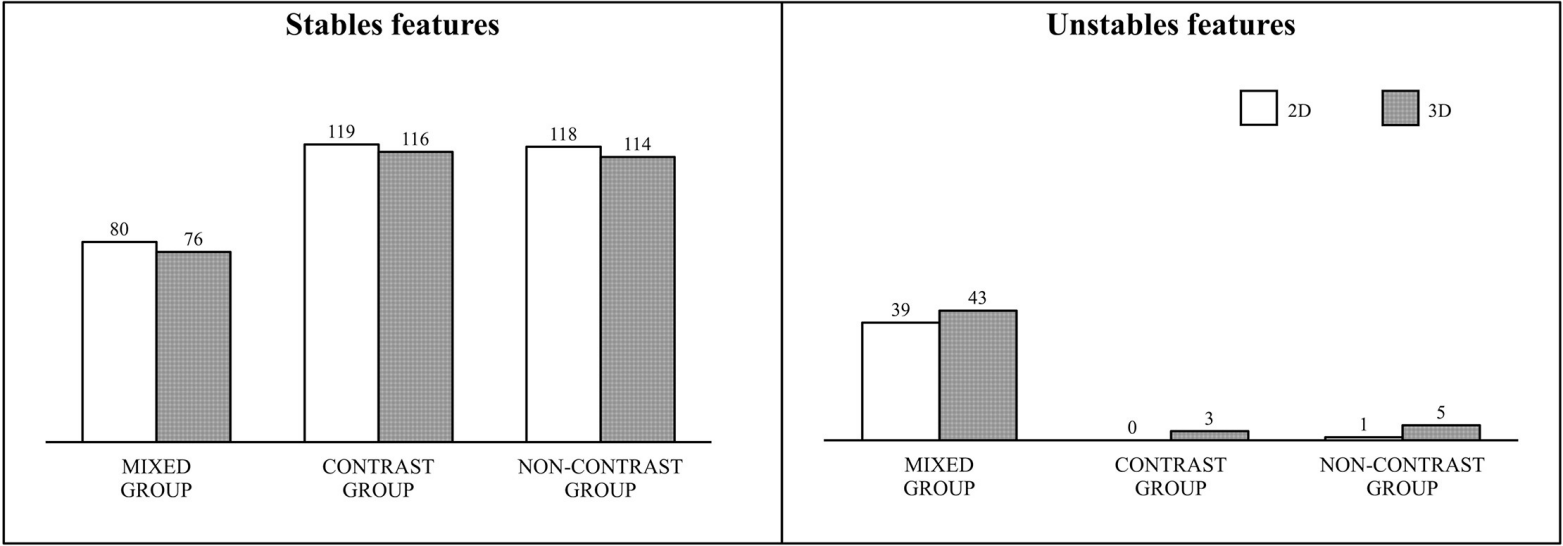
Stability of the radiomic features for the three groups assessed. Features were divided in 2D or in 3D.

As shown in Fig 3, 2D features proved to be more stable then 3D features on contrast-enhanced and non-contrast enhanced CT images of patients with OC.

Table 3 summarizes the results obtained after comparing the corresponding 2D and 3D stable features with the paired two-sided Wilcoxon signed rank test. Among the 76 remaining corresponding stable features in the mixed group, only 6 features did not show a statistically significant difference if obtained in 2D or in 3D. For the contrast group, 15 stable features were not statistically different if extracted in 2D or 3D. The greatest number of stable features (n = 17) that were independent if computed slice-by-slice or from a volume was found in the non-contrast group. Four independent features (inverse variance_GLCM_, large distance emphasis_GLDZM_, zone distance non uniformity norm_GLDZM_, zone distance variance_GLDZM_) were stable across dimensionality and contrast administration in the three groups considered. Among them, inverse variance_GLCM_ was the only feature that also showed to be independent from the type of aggregation methods used. Comparison of the inverse variance_GLCM_ obtained with merging and without merging showed a high correlation (ICC > 0.98). Due to this result, the two features were clustered and inverse variance_GLCM_ obtained without merging was used as leader of the set in the following analysis.

**Table 3:** List of stable radiomic features not statistically different if extracted using one segment layer at a time or considering the whole tumour volume in CT images of the oesophagus. In bold, common features among three cohorts considered that showed to be stable and dimensionality and contrast agent independent. GLCM, grey level co-occurrence matrix; GLRLM, grey level run length matrix; GLSZM, grey level size zone matrix; GLDZM, grey level distance zone matrix; NGTDM, neighbourhood grey tone difference matrix; *, feature computed with merging.

Texture type	Feature	Mixed group	Contrast group	Non-contrast group
GLCM	contrast			yes
	***Inverse variance***	yes	yes	yes
	Inverse variance*	yes	yes	yes
GLRL	High GL run emp		yes	yes
	Long run low GL emp		yes	yes
	RL non uniformity norm		yes	yes
	Run percentage		yes	yes
	RL variance		yes	yes
	High GL run emp*		yes	yes
	Long run low GL emp*		yes	yes
	Run percentage*		yes	yes
	RL variance*		yes	yes
GLSZM	Small zone emphasis		yes	yes
GLDZM	Small distance emphasis	yes		yes
	***Large distance emphasis***	yes	yes	yes
	***Zone distance non uniformity norm***	yes	yes	yes
	***Zone distance variance***	yes	yes	yes

The Cox regression model was constructed using 5 clinical variables (age, sex, TNM stage, WHO performance status and contrast) and 4 radiomic variables (inverse variance_GLCM_, large distance emphasis_GLDZM_, zone distance non uniformity norm_GLDZM_, zone distance variance_GLDZM_). At the final step of the prognostic model, two variables were found significantly associated with overall survival: TNM stage (p-value = 0.017, HR = 1.48, 95% CI = 1.07–2.05) and zone distance variance_GLDZM_ (p-value = 0.032, HR = 1.25, 95% CI = 1.02–1.52). Since TNM stage is already known as a prognostic marker in oesophageal cancer^8^, only the prognostic significance of zone distance variance_GLDZM_ was explored further.

**Fig 4:**
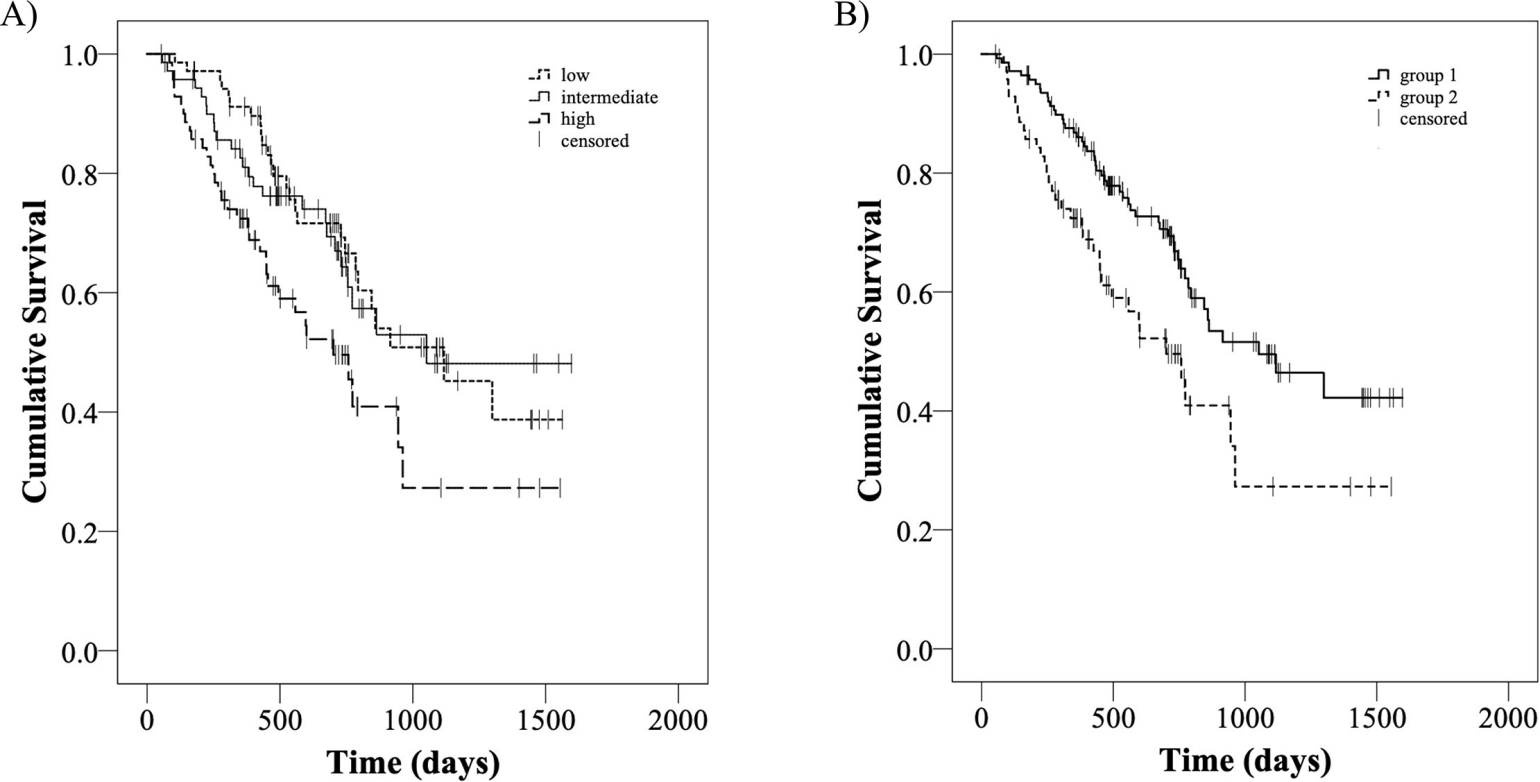
Kaplan-Meier survival curves of the cohort used in this work. Patients divided in a) low-risk group (zone distance varianceGLDZM from 0.15 to 0.85), intermediate-risk group (zone distance varianceGLDZM from 0.86 to 1.69) and high-risk group (zone distance varianceGLDZM from 1.70 to 6.2) based on the prognostic score and b) group 1 (zone distance varianceGLDZM < 1.70) and group 2 (zone distance varianceGLDZM = > 1.70) considering a threshold of zone distance varianceGLDZM. There was a significant difference in OS when dividing in low, intermediate and high-risk group (X2 = 7.37, df = 1, p-value = 0.007) or when using a threshold of zone distance varianceGLDZM (X2 = 7.692, df = 1, p-value = 0.006).

Zone distance variance_GLDZM_ was found to be correlated with an increased chance of mortality and was used as a prognostic score to separate patients into three groups each populated with 71 subjects. The score ranges used for separating the cohort were the following: for group 1, labelled as low-risk group, from 0.15 to 0.85, for group 2, labelled as intermediate-risk group, from 0.86 to 1.69 and for group 3, labelled as high-risk group, from 1.70 to 6.2. The median OS of each low, intermediate and high-risk group was 688 days (95% CI 597.4–773.4), 554 days (95% CI 532.9–700.9), 436.5 days (95% CI 427–580.9), respectively. The log-rank test determined a significant difference (X^2^ = 7.767, df = 2, p-value = 0.021) as shown in Fig 4A.

The significant difference (X^2^ = 7.692, df = 1, p-value = 0.006) also persisted when merging the low and the intermediate groups. Fig 4B shows the risk stratification when dividing the cohort of patients in two groups by considering a threshold of zone distance variance_GLDZM_ equal to 1.70.

## 4. Discussion

In this study, we evaluated the potential additional value derived from radiomic features extracted from contrast and non-contrast enhanced CT images of a RCT in the development of a new prognostic model in OC. In particular, we investigated: 1) the relationship between CT textures and the administration of I.V. contrast; 2) stability of CT features analysis when computed in 2D or from a 3D volume in homogeneous and inhomogeneous OC cohorts; and 3) the prognostic value of stable CT features in current OC staging systems. To the best of our knowledge, this is the first study to develop a prognostic model with stable and dimensionality-independent features using clinical RCT trial data of contrast and non-contrast enhanced CT images of OC patients.

Our analysis showed a statistical dependency of radiomics features extracted from CT scans on I.V. contrast medium. As expected, radiomic features are more stable if extracted from homogeneous cohorts. Furthermore, our study showed that most of the stable features extracted from CT images acquired after the administration of I.V. contrast are also stable when obtained from cohort of patients scanned without I.V. contrast. This is in line with the recent work of Badic et al. [24], in which the link between features extracted from contrast enhanced and non-contrast enhanced CT images of primary colorectal cancer of 61 patients was investigated. However, a comparison with our findings was not possible because even though a large number of metrics were computed (first-order, second-order and third-order), no GLDZM-based texture analysis was performed.

We also further investigated differences when radiomic features are extracted in 2D or in 3D. In line with the work of Shen et al. [25], our results have shown that 2D features performed slightly better than the corresponding 3D ones in the three cohorts considered. In particular, no 2D features were unstable when CT images were acquired with I.V. contrast and only one was found unstable in the non-contrast group. In general, the number of unstable 3D features was slightly higher than the number of unstable 2D features. This could be explained by different voxel sizes of the CT images acquired in this multi-centre study (axial resolution range: 0.71–1.37 mm; slice thickness range: 2.5–5 mm). The dependency of radiomic features on CT voxel size has already been investigated in several studies [26, 27]. However, the production of scans reconstructed to different voxel resolution is frequent in multi-centre trials and even the implementation of an image resampling method before radiomic feature extraction may not warrant the complete removal of voxel size dependency. In fact, Mackin et al. [28] showed that image resampling alone tends to increase the variability of radiomics features extracted from CT images of lung cancer patients. They also suggested that the variability can be reduced with a harmonization the pixel size based on scan resampling in the time domain and a Butterworth low-pass filtering in the frequency domain.

In general, 2D features extracted from CT images of OC patients performed slightly better than 3D ones when evaluated in terms of stability, dimensionality and I.V. contrast dependency. Although, 3D features might carry more information and allow to perform a whole tumour analysis [29], they are affected by the slice thickness of the imaging data [30] that is typically higher than the in-plane resolution [31]. However, based on the results of this study we cannot recommend 2D over 3D features for clinical research and radiomics studies. Future investigations should consider including the stable features identified in this work. The list of stable features is reported in S1 Table.

Analysis of dimensionality and contrast-independent stable features with the Cox regression method identified zone distance variance_GLDZM_ as the only radiomic feature statistically correlated with OS. GLDZM evaluates the relation between grey levels and location by counting the number of groups (or zones) of voxels with a specific discretised grey level value and the same distance to the edge of the ROI considered. In particular, zone distance variance_GLDZM_ estimates the variance in zone counts for the different zone distances which provides a quantitative characterization of tumour heterogeneity. This is known to correlate with poor prognosis [32]. In agreement with the model, zone distance variance_GLDZM_ is significantly associated with overall survival. Patients with increased zone distance variance_GLDZM_ have a higher hazard ratio and a therefore shorter life expectancy. In particular, we have identified a high-risk group above a threshold 1.7 for zone distance variance_GLDZM_. This result confirms the potential prognostic value of textural features measuring tumour heterogeneity as already shown by different radiomic studies in esophageal [33], lungs [34], head and neck cancer [35]. This imaging biomarker needs to be tested in a validation dataset to prove its prognostic value.

This study has some limitations. Although the great strength of the study is that the data was obtained from multiple centres in the context of a randomised control trial, validation of the model constructed using stable features was not performed. According to the TRIPOD scheme, this study can be classified as a type 1 study in which the development of a prediction model and the predictive performance are evaluated using the same data. The dataset used in this work was not split in training and validation set to power the prognostic model appropriately by reducing as much as possible the rate of false positives (type-I error) and by improving the ability to detect a true difference between groups (type-II error) [36]. This ensure a high stability of the model and its transferability and applicability to a wider oesophageal cancer population independent to contrast administration. Second, the robustness of the extracted radiomic features in terms of reproducibility and repeatability in contrast and non-contrast enhanced CT images of OC patients could not be investigated because of the design of the RCT. Future work will address these limitations by externally validating the prognostic model and by evaluating the robustness of radiomic features obtained from CT images in different multi-centre datasets.

## 5. Conclusion

We investigated the stability, dimensionality and contrast agent dependency of radiomic features extracted from contrasted and non-contrast CT examinations of patients with OC, acquired during the multi-centre SCOPE1 RCT. Zone distance variance_GLDZM_ was identified as the only stable CT radiomic feature statistically correlated with overall survival, independent of dimensionality and contrast administration. This feature was able to identify high-risk patients and if validated, could be the subject of a future clinical trial aiming to improve clinical decision making and personalise OC treatment.

## Supporting information

S1 Table List of 2D and 3D radiomic features that showed to be stable when extracted from the mixed, the contrast and the non-contrast group. GLCM, grey level co-occurrence matrix; GLRLM, grey level run length matrix; GLSZM, grey level size zone matrix; GLDZM, grey level distance zone matrix; NGTDM, neighbourhood grey tone difference matrix; *, feature computed with merging.
(DOCX)

## Abbreviations

CRT: Chemo-radiotherapy
IBSI: Image biomarker standardisation initiative
GLCM: Grey level co-occurrence matrix
GLDZM: Grey level distance zone matrix
GLRL: Grey level run length matrix
GLSZM: Grey level size zone matrix
GTV: Gross tumour volume
ICC: Intraclass correlation coefficient
I.V.: Intravenous
NGTDM: Neighbourhood grey tone difference matrix
OC: Oesophageal cancer
OS: Overall survival
RCT: Randomised controlled trial
TNM: tumour, node and metastasis
