# Supplementary material for: Discovery of stable and prognostic CT-based radiomic features independent of contrast administration and dimensionality in oesophageal cancer
Source: PLoS One. 2019 Nov 22;14(11):e0225550. doi: 10.1371/journal.pone.0225550 (PMC6874382; doi:10.1371/journal.pone.0225550)
Supplement: S1 Table — GLCM, grey level co-occurrence matrix; GLRLM, grey level run length matrix; GLSZM, grey level size zone matrix; GLDZM, grey level distance zone matrix; NGTDM, neighbourhood grey tone difference matrix; *, feature computed with merging. (DOCX) [file pone.0225550.s001.docx]

**Discovery of stable and prognostic CT-based radiomic features independent of contrast administration and dimensionality in oesophageal cancer**

Concetta Piazzese^1,2*^, Kieran Foley^2^, Philip Whybra^1^, Chris Hurt^3^, Tom Crosby^2^, Emiliano Spezi^1,2^

^1^ School of Engineering, Cardiff University, Cardiff, United Kingdom

^2^ Velindre Cancer Centre, Cardiff, United Kingdom

^3^ Centre for Trials Research, Cardiff, United Kingdom

Supplementary Information

*** Corresponding author**

E-mail: [concettap@cardiff.ac.uk](mailto:concettap@cardiff.ac.uk) (CP)

**S1 Table. List of 2D and 3D radiomic features that showed to be stable when extracted from the mixed, the contrast and the non-contrast group.** GLCM, grey level co-occurrence matrix; GLRLM, grey level run length matrix; GLSZM, grey level size zone matrix; GLDZM, grey level distance zone matrix; NGTDM, neighbourhood grey tone difference matrix; *, feature computed with merging.

| Texture type | Feature | Mixed group | Contrast group | Non-contrast group |
| --- | --- | --- | --- | --- |
| GLCM | $\text{Joint maximum}_{\text{2D}}$ | no | yes | yes |
|  | Joint maximum_3D_ | no | yes | yes |
|  | $\text{Joint maximum}_{\text{2D}}^{\text{*}}$ | no | yes | yes |
|  | $\text{Joint maximum}_{\text{3D}}^{\text{*}}$ | no | yes | yes |
|  | $\text{Joint average}_{\text{2D}}$ | no | yes | yes |
|  | $\text{Joint average}_{\text{3D}}$ | no | yes | yes |
|  | $\text{Joint average}_{\text{2D}}^{\text{*}}$ | no | yes | yes |
|  | $\text{Joint average}_{\text{3D}}^{\text{*}}$ | no | yes | yes |
|  | $\text{Joint variance}_{\text{2D}}$ | yes | yes | yes |
|  | $\text{Joint variance}_{\text{3D}}$ | yes | yes | yes |
|  | $\text{Joint variance}_{\text{2D}}^{\text{*}}$ | yes | yes | yes |
|  | $\text{Joint variance}_{\text{3D}}^{\text{*}}$ | yes | yes | yes |
|  | $\text{Joint entropy}_{\text{2D}}$ | yes | yes | yes |
|  | $\text{Joint entropy}_{\text{3D}}$ | yes | yes | yes |
|  | $\text{Joint entropy}_{\text{2D}}^{\text{*}}$ | yes | yes | yes |
|  | $\text{Joint entropy}_{\text{3D}}^{\text{*}}$ | yes | yes | yes |
|  | $\text{Difference average}_{\text{2D}}$ | yes | yes | yes |
|  | $\text{Difference average}_{\text{3D}}$ | yes | yes | yes |
|  | $\text{Difference average}_{\text{2D}}^{\text{*}}$ | yes | yes | yes |
|  | $\text{Difference average}_{\text{3D}}^{\text{*}}$ | yes | yes | yes |
|  | $\text{Difference variance}_{\text{2D}}$ | yes | yes | yes |
|  | $\text{Difference variance}_{\text{3D}}^{\text{*}}$ | yes | yes | yes |
|  | $\text{Difference entropy}_{\text{2D}}$ | yes | yes | yes |

S1 Table. (continued)

| Texture type | Feature | Mixed group | Contrast group | Non-contrast group |
| --- | --- | --- | --- | --- |
| GLCM | $\text{Difference entropy}_{\text{3D}}$ | yes | yes | yes |
|  | $\text{Difference entropy}_{\text{2D}}^{\text{*}}$ | yes | yes | yes |
|  | $\text{Difference entropy}_{\text{3D}}^{\text{*}}$ | yes | yes | yes |
|  | $\text{Sum average}_{\text{2D}}$ | no | yes | yes |
|  | $\text{Sum average}_{\text{3D}}$ | no | yes | yes |
|  | $\text{Sum average}_{\text{2D}}^{\text{*}}$ | no | yes | yes |
|  | $\text{Sum average}_{\text{3D}}^{\text{*}}$ | no | yes | yes |
|  | $\text{Sum variance}_{\text{2D}}$ | yes | yes | yes |
|  | $\text{Sum variance}_{\text{3D}}$ | yes | yes | yes |
|  | $\text{Sum variance}_{\text{2D}}^{\text{*}}$ | yes | yes | yes |
|  | $\text{Sum variance}_{\text{3D}}^{\text{*}}$ | yes | yes | yes |
|  | $\text{Sum entropy}_{\text{2D}}$ | yes | yes | yes |
|  | $\text{Sum entropy}_{\text{3D}}$ | yes | yes | yes |
|  | $\text{Sum entropy}_{\text{2D}}^{\text{*}}$ | yes | yes | yes |
|  | $\text{Sum entropy}_{\text{3D}}^{\text{*}}$ | yes | yes | yes |
|  | $\text{Angular second moment}_{\text{2D}}$ | no | yes | yes |
|  | $\text{Angular second moment}_{\text{3D}}$ | no | yes | yes |
|  | $\text{Angular second moment}_{\text{2D}}^{\text{*}}$ | no | yes | yes |
|  | $\text{Angular second moment}_{\text{3D}}^{\text{*}}$ | no | yes | yes |
|  | $\text{Contrast}_{\text{2D}}$ | yes | yes | yes |
|  | $\text{Contrast}_{\text{3D}}$ | yes | yes | yes |
|  | $\text{Contrast}_{\text{2D}}^{\text{*}}$ | yes | yes | yes |
|  | $\text{Contrast}_{\text{3D}}^{\text{*}}$ | yes | yes | yes |

S1 Table. (continued)

| Texture type | Feature | Mixed group | Contrast group | Non-contrast group |
| --- | --- | --- | --- | --- |
| GLCM | $\text{Dissimilarity}_{\text{2D}}$ | yes | yes | yes |
|  | $\text{Dissimilarity}_{\text{3D}}$ | yes | yes | yes |
|  | $\mathrm{Dissimilarity}_{\text{2D}}^{\text{*}}$ | yes | yes | yes |
|  | $\mathrm{Dissimilarity}_{\text{3D}}^{\text{*}}$ | yes | yes | yes |
|  | $\text{Inverse difference}_{\text{2D}}$ | no | yes | yes |
|  | $\text{Inverse difference}_{\text{3D}}$ | no | yes | yes |
|  | ${Inverse difference}_{\text{2D}}^{\text{*}}$ | no | yes | yes |
|  | ${Inverse difference}_{\text{3D}}^{\text{*}}$ | no | yes | yes |
|  | $\text{Inverse difference norm}_{\text{2D}}$ | yes | yes | yes |
|  | $\text{Inverse difference norm}_{\text{3D}}$ | yes | yes | yes |
|  | $\text{Inverse difference norm}_{\text{2D}}^{\text{*}}$ | yes | yes | yes |
|  | $\text{Inverse difference norm}_{\text{3D}}^{\text{*}}$ | yes | yes | yes |
|  | $\text{Inverse diff moment}_{\text{2D}}$ | no | yes | yes |
|  | $\text{Inverse diff moment}_{\text{3D}}$ | no | yes | yes |
|  | ${Inverse diff moment}_{\text{2D}}^{\text{*}}$ | no | yes | yes |
|  | ${Inverse diff moment}_{\text{3D}}^{\text{*}}$ | no | yes | yes |
|  | $\text{Inverse diff moment norm}_{\text{2D}}$ | yes | yes | yes |
|  | $\text{Inverse diff moment norm}_{\text{3D}}$ | yes | yes | yes |
|  | $\text{Inverse diff moment norm}_{\text{2D}}^{\text{*}}$ | yes | yes | yes |
|  | $\text{Inverse diff moment norm}_{\text{3D}}^{\text{*}}$ | yes | yes | yes |
|  | $\text{Inverse invariance}_{\text{2D}}$ | yes | yes | yes |
|  | $\text{Inverse invariance}_{\text{3D}}$ | yes | yes | yes |
|  | $\text{Inverse variance}_{\text{2D}}^{\text{*}}$ | yes | yes | yes |

S1 Table. (continued)

| Texture type | Feature | Mixed group | Contrast group | Non-contrast group |
| --- | --- | --- | --- | --- |
| GLCM | $\text{Inverse variance}_{\text{3D}}^{\text{*}}$ | yes | yes | yes |
|  | $\text{Autocorrelation}_{\text{2D}}$ | no | yes | yes |
|  | $\text{Autocorrelation}_{\text{3D}}$ | no | yes | yes |
|  | $\text{Autocorrelation}_{\text{2D}}^{\text{*}}$ | no | yes | yes |
|  | $\text{Autocorrelation}_{\text{3D}}^{\text{*}}$ | no | yes | yes |
|  | $\text{Correlation}_{\text{2D}}$ | yes | yes | yes |
|  | $\text{Correlation}_{\text{3D}}$ | yes | yes | yes |
|  | $\text{Correlation}_{\text{2D}}^{\text{*}}$ | yes | yes | yes |
|  | $\text{Correlation}_{\text{3D}}^{\text{*}}$ | yes | yes | yes |
|  | $\text{Cluster tendency}_{\text{2D}}$ | yes | yes | yes |
|  | $\text{Cluster tendency}_{\text{3D}}$ | yes | yes | yes |
|  | $\text{Cluster tendency}_{\text{2D}}^{\text{*}}$ | yes | yes | yes |
|  | $\text{Cluster tendency}_{\text{3D}}^{\text{*}}$ | yes | yes | yes |
|  | $\text{Cluster shade}_{\text{2D}}$ | yes | yes | yes |
|  | $\text{Cluster shade}_{\text{3D}}$ | yes | yes | yes |
|  | $\text{Cluster shade}_{\text{2D}}^{\text{*}}$ | yes | yes | yes |
|  | $\text{Cluster shade}_{\text{3D}}^{\text{*}}$ | yes | yes | yes |
|  | $\text{Cluster prominence}_{\text{2D}}$ | yes | yes | yes |
|  | $\text{Cluster prominence}_{\text{3D}}$ | yes | yes | yes |
|  | $\text{Cluster prominence}_{\text{2D}}^{\text{*}}$ | yes | yes | yes |
|  | $\text{Cluster prominence}_{\text{3D}}^{\text{*}}$ | yes | yes | yes |
|  | $\text{Info correlation 1}_{\text{2D}}$ | yes | yes | yes |
|  | $\text{Info correlation 1}_{\text{3D}}$ | yes | yes | yes |

S1 Table. (continued)

| Texture type | Feature | Mixed group | Contrast group | Non-contrast group |
| --- | --- | --- | --- | --- |
| GLCM | $\text{Info correlation 1}_{\text{2D}}^{\text{*}}$ | yes | yes | yes |
|  | $\text{Info correlation 1}_{\text{3D}}^{\text{*}}$ | yes | yes | yes |
|  | $\text{Info correlation 2}_{\text{2D}}$ | yes | yes | yes |
|  | $\text{Info correlation 2}_{\text{3D}}$ | yes | yes | yes |
|  | $\text{Info correlation 2}_{\text{2D}}^{\text{*}}$ | yes | yes | yes |
|  | $\text{Info correlation 2}_{\text{3D}}^{\text{*}}$ | yes | yes | yes |
| GLRL | $\text{Short run emp}_{\text{2D}}$ | no | yes | yes |
|  | $\text{Short run emp}_{\text{3D}}$ | no | yes | yes |
|  | $\text{Short run emp}_{\text{2D}}^{\text{*}}$ | no | yes | yes |
|  | $\text{Short run emp}_{\text{3D}}^{\text{*}}$ | no | yes | yes |
|  | $\text{Long run emp}_{\text{2D}}$ | no | yes | yes |
|  | $\text{Long run emp}_{\text{3D}}$ | no | yes | yes |
|  | $\text{Long run emp}_{\text{2D}}^{\text{*}}$ | no | yes | yes |
|  | $\text{Long run emp}_{\text{3D}}^{\text{*}}$ | no | yes | yes |
|  | $\text{Low GL run emp}_{\text{2D}}$ | no | yes | yes |
|  | $\text{Low GL run emp}_{\text{3D}}$ | no | yes | yes |
|  | $\text{Low GL run emp}_{\text{2D}}^{\text{*}}$ | yes | yes | yes |
|  | $\text{Low GL run emp}_{\text{3D}}^{\text{*}}$ | no | yes | yes |
|  | $\text{High GL run emp}_{\text{2D}}$ | no | yes | yes |
|  | $\text{High GL run emp}_{\text{3D}}$ | no | yes | yes |
|  | $\text{High GL run emp}_{\text{2D}}^{\text{*}}$ | no | yes | yes |
|  | $\text{High GL run emp}_{\text{3D}}^{\text{*}}$ | no | yes | yes |
|  | $\text{Short run low GL emp}_{\text{2D}}$ | yes | yes | yes |

S1 Table. (continued)

| Texture type | Feature | Mixed group | Contrast group | Non-contrast group |
| --- | --- | --- | --- | --- |
| GLRL | $\text{Short run low GL emp}_{\text{3D}}$ | yes | yes | yes |
|  | $\text{Short run low GL emp}_{\text{2D}}^{\text{*}}$ | yes | yes | yes |
|  | $\text{Short run low GL emp}_{\text{3D}}^{\text{*}}$ | yes | yes | yes |
|  | $\text{Short run high GL emp}_{\text{2D}}$ | no | yes | yes |
|  | $\text{Short run high GL emp}_{\text{3D}}$ | no | yes | no |
|  | $\text{Short run high GL emp}_{\text{2D}}^{\text{*}}$ | no | yes | no |
|  | $\text{Short run high GL emp}_{\text{3D}}^{\text{*}}$ | no | yes | no |
|  | $\text{Long run low GL emp}_{\text{2D}}$ | no | yes | yes |
|  | $\text{Long run low GL emp}_{\text{3D}}$ | no | yes | yes |
|  | $\text{Long run low GL emp}_{\text{2D}}^{\text{*}}$ | no | yes | yes |
|  | $\text{Long run low GL emp}_{\text{3D}}^{\text{*}}$ | no | yes | yes |
|  | $\text{Long run high GL emp}_{\text{2D}}$ | yes | yes | yes |
|  | $\text{Long run high GL emp}_{\text{3D}}$ | yes | yes | yes |
|  | $\text{Long run high GL emp}_{\text{2D}}^{\text{*}}$ | yes | yes | yes |
|  | $\text{Long run high GL emp}_{\text{3D}}^{\text{*}}$ | yes | yes | yes |
|  | $\text{GL non uniformity}_{\text{2D}}$ | yes | yes | yes |
|  | $\text{GL non uniformity}_{\text{3D}}$ | yes | yes | yes |
|  | $\text{GL non uniformity}_{\text{2D}}^{\text{*}}$ | yes | yes | yes |
|  | $\text{GL non uniformity}_{\text{3D}}^{\text{*}}$ | yes | yes | yes |
|  | $\text{GL non uniformity norm}_{\text{2D}}$ | yes | yes | yes |
|  | $\text{GL non uniformity norm}_{\text{3D}}$ | yes | yes | yes |
|  | $\text{GL non uniformity norm}_{\text{2D}}^{\text{*}}$ | yes | yes | yes |
|  | $\text{GL non uniformity norm}_{\text{2D}}^{\text{*}}$ | yes | yes | yes |

S1 Table. (continued)

| Texture type | Feature | Mixed group | Contrast group | Non-contrast group |
| --- | --- | --- | --- | --- |
| GLRL | $\text{RL non uniformity}_{\text{2D}}$ | yes | yes | yes |
|  | $\text{RL non uniformity}_{\text{3D}}$ | yes | yes | yes |
|  | $\text{RL non uniformity}_{\text{2D}}^{\text{*}}$ | yes | yes | yes |
|  | $\text{RL non uniformity}_{\text{3D}}^{\text{*}}$ | yes | yes | yes |
|  | $\text{RL non uniformity norm}_{\text{2D}}$ | no | yes | yes |
|  | $\text{RL non uniformity norm}_{\text{3D}}$ | no | yes | yes |
|  | $\text{RL non uniformity norm}_{\text{2D}}^{\text{*}}$ | no | yes | yes |
|  | $\text{RL non uniformity norm}_{\text{3D}}^{\text{*}}$ | no | yes | yes |
|  | $\text{Run percentage}_{\text{2D}}$ | no | yes | yes |
|  | $\text{Run percentage}_{\text{3D}}$ | no | yes | yes |
|  | $\text{Run percentage}_{\text{2D}}^{\text{*}}$ | no | yes | yes |
|  | $\text{Run percentage}_{\text{3D}}^{\text{*}}$ | no | yes | yes |
|  | $\text{GL variance}_{\text{2D}}$ | yes | yes | yes |
|  | $\text{GL variance}_{\text{3D}}$ | yes | yes | yes |
|  | $\text{GL variance}_{\text{2D}}^{\text{*}}$ | yes | yes | yes |
|  | $\text{GL variance}_{\text{3D}}^{\text{*}}$ | yes | yes | yes |
|  | $\text{RL variance}_{\text{2D}}$ | no | yes | yes |
|  | $\text{RL variance}_{\text{3D}}$ | no | yes | yes |
|  | $\text{RL variance}_{\text{2D}}^{\text{*}}$ | no | yes | yes |
|  | $\text{RL variance}_{\text{3D}}^{\text{*}}$ | no | yes | yes |
|  | $\text{Run entropy}_{\text{2D}}$ | yes | yes | yes |
|  | $\text{Run entropy}_{\text{3D}}$ | yes | yes | yes |
|  | $\text{Run entropy}_{\text{2D}}^{\text{*}}$ | yes | yes | yes |

S1 Table. (continued)

| Texture type | Feature | Mixed group | Contrast group | Non-contrast group |
| --- | --- | --- | --- | --- |
| GLRL | $\text{Run entropy}_{\text{3D}}^{\text{*}}$ | yes | yes | yes |
| GLSZM | $\text{Small zone emphasis}_{\text{2D}}$ | yes | yes | yes |
|  | $\text{Small zone emphasis}_{\text{3D}}$ | no | yes | yes |
|  | $\text{Large zone emphasis}_{\text{2D}}$ | no | yes | yes |
|  | $\text{Large zone emphasis}_{\text{3D}}$ | no | yes | yes |
|  | $\text{Low GL zone emphasis}_{\text{2D}}$ | yes | yes | yes |
|  | $\text{Low GL zone emphasis}_{\text{3D}}$ | yes | yes | yes |
|  | $\text{High GL zone emphasis}_{\text{2D}}$ | no | yes | yes |
|  | $\text{High GL zone emphasis}_{\text{3D}}$ | no | yes | no |
|  | $\text{Small zone low GL emphasis}_{\text{2D}}$ | yes | yes | yes |
|  | $\text{Small zone low GL emphasis}_{\text{3D}}$ | yes | yes | yes |
|  | $\text{Small zone high GL emphasis}_{\text{2D}}$ | no | yes | yes |
|  | $\text{Small zone high GL emphasis}_{\text{2D}}$ | no | yes | yes |
|  | $\text{Large zone low GL emphasis}_{\text{2D}}$ | no | yes | yes |
|  | $\text{Large zone low GL emphasis}_{\text{3D}}$ | no | yes | yes |
|  | $\text{Large zone high GL emphasis}_{\text{2D}}$ | yes | yes | yes |
|  | $\text{Large zone high GL emphasis}_{\text{3D}}$ | yes | yes | yes |
|  | $\text{GL non uniformity}_{\text{2D}}$ | yes | yes | yes |
|  | $\text{GL non uniformity}_{\text{3D}}$ | yes | no | yes |
|  | $\text{GL non uniformity norm}_{\text{2D}}$ | yes | yes | yes |
|  | $\text{GL non uniformity norm}_{\text{3D}}$ | yes | yes | yes |
|  | $\text{Zone size non uniformity}_{\text{2D}}$ | yes | yes | yes |
|  | $\text{Zone size non uniformity}_{\text{3D}}$ | yes | yes | yes |

S1 Table. (continued)

| Texture type | Feature | Mixed group | Contrast group | Non-contrast group |
| --- | --- | --- | --- | --- |
| GLSZM | $\text{Size zone non uniformity norm}_{\text{2D}}$ | yes | yes | yes |
|  | $\text{Size zone non uniformity norm}_{\text{3D}}$ | no | yes | yes |
|  | $\text{Zone percentage}_{\text{2D}}$ | no | yes | yes |
|  | $\text{Zone percentage}_{\text{3D}}$ | yes | yes | yes |
|  | $\text{GL variance}_{\text{2D}}$ | yes | yes | yes |
|  | $\text{GL variance}_{\text{3D}}$ | yes | yes | yes |
|  | $\text{Zone size variance}_{\text{2D}}$ | no | yes | yes |
|  | $\text{Zone size variance}_{\text{3D}}$ | no | yes | yes |
|  | $\text{Zone size entropy}_{\text{2D}}$ | yes | yes | yes |
|  | $\text{Zone size entropy}_{\text{3D}}$ | yes | yes | yes |
| GLDZM | $\text{Small distance emphasis}_{\text{2D}}$ | yes | yes | yes |
|  | $\text{Small distance emphasis}_{\text{3D}}$ | yes | yes | yes |
|  | $\text{Large distance emphasis}_{\text{2D}}$ | yes | yes | yes |
|  | $\text{Large distance emphasis}_{\text{3}\text{D}}$ | yes | yes | yes |
|  | $\text{Low grey level zone emphasis}_{\text{2D}}$ | yes | yes | yes |
|  | $\text{Low grey level zone emphasis}_{\text{3D}}$ | yes | yes | yes |
|  | $\text{High grey level zone emphasis}_{\text{2D}}$ | no | yes | yes |
|  | $\text{High grey level zone emphasis}_{\text{3D}}$ | no | yes | no |
|  | $\text{Small distance low grey L Emphasis}_{\text{2D}}$ | yes | yes | yes |
|  | $\text{Small distance low grey L Emphasis}_{\text{3D}}$ | yes | yes | yes |
|  | $\text{Small distance high grey L Emphasis}_{\text{2D}}$ | yes | yes | yes |
|  | $\text{Small distance high grey L Emphasis}_{\text{3D}}$ | no | yes | no |
|  | $\text{Large distance low grey L Emphasis}_{\text{2D}}$ | yes | yes | yes |

S1 Table. (continued)

| Texture type | Feature | Mixed group | Contrast group | Non-contrast group |
| --- | --- | --- | --- | --- |
| GLDZM | $\text{Large distance low grey L Emphasis}_{\text{3D}}$ | yes | yes | yes |
|  | ${\text{Large distance }\text{high}\text{ grey L Emphasis}}_{\text{2D}}$ | yes | yes | yes |
|  | ${\text{Large distance }\text{high}\text{ grey L Emphasis}}_{\text{3D}}$ | yes | yes | yes |
|  | $\text{Grey level non uniformity}_{\text{2D}}$ | yes | yes | yes |
|  | $\text{Grey level non uniformity}_{\text{3D}}$ | yes | no | yes |
|  | $\text{Grey level non uniformity norm}_{\text{2D}}$ | yes | yes | yes |
|  | $\text{Grey level non uniformity norm}_{\text{3D}}$ | yes | yes | yes |
|  | $\text{Zone distance non uniformity}_{\text{2D}}$ | yes | yes | yes |
|  | $\text{Zone distance non uniformity}_{\text{3D}}$ | yes | no | yes |
|  | $\text{Zone distance non uniformity norm}_{\text{2D}}$ | yes | yes | yes |
|  | $\text{Zone distance non uniformity norm}_{\text{3D}}$ | yes | yes | yes |
|  | $\text{Zone percentage}_{\text{2D}}$ | no | yes | yes |
|  | $\text{Zone percentage}_{\text{3D}}$ | yes | yes | yes |
|  | $\text{Grey level variance}_{\text{2D}}$ | yes | yes | yes |
|  | $\text{Grey level variance}_{\text{3D}}$ | yes | yes | yes |
|  | $\text{Zone distance variance}_{\text{2D}}$ | yes | yes | yes |
|  | $\text{Zone distance variance}_{\text{3D}}$ | yes | yes | yes |
|  | $\text{Zone distance entropy}_{\text{2D}}$ | yes | yes | yes |
|  | $\text{Zone distance entropy}_{\text{3D}}$ | yes | yes | yes |
| NGTDM | $\text{Coarseness}_{\text{2D}}$ | yes | yes | yes |
|  | $\text{Coarseness}_{\text{3D}}$ | yes | yes | yes |
|  | $\text{Contrast}_{\text{2D}}$ | yes | yes | yes |
|  | $\text{Contrast}_{\text{3D}}$ | yes | yes | yes |

S1 Table. (continued)

| Texture type | Feature | Mixed group | Contrast group | Non-contrast group |
| --- | --- | --- | --- | --- |
| NGTDM | $\text{Busyness}_{\text{2D}}$ | yes | yes | yes |
|  | $\text{Busyness}_{\text{3D}}$ | no | yes | yes |
|  | $\text{Complexity}_{\text{2D}}$ | yes | yes | yes |
|  | $\text{Complexity}_{\text{3}\text{D}}$ | yes | yes | yes |
|  | $\text{Strength}_{\text{2D}}$ | yes | yes | yes |
|  | $\text{Strength}_{\text{3}\text{D}}$ | no | yes | yes |
